# Risk Assessment of Dibutyl Phthalate (DBP) and Bis(2-Ethylhexyl) Phthalate (DEHP) in Hot Pot Bases with a Hybrid Modeling Approach

**DOI:** 10.3390/toxics14020150

**Published:** 2026-02-02

**Authors:** Xiangyu Bian, Siyu Huang, Dongya Chen, Depeng Jiang, Daoyuan Yang, Yingzi Zhao, Zhujun Liu, Shiqi Chen, Yan Song, Haixia Sui, Jinfang Sun

**Affiliations:** 1 Department of Epidemiology and Biostatistics, School of Public Health, Southeast University, Nanjing 210000, China; 2 Key Laboratory of Environmental Medicine Engineering, Ministry of Education, School of Public Health, Southeast University, Nanjing 210000, China; 3 Key Laboratory of Condiment Supervision Technology, State Administration for Market Regulation, Chongqing Institute for Food and Drug Control, Chongqing 401121, China; 4 Jiangsu Provincial Center for Disease Control and Prevention, 172 Jiangsu Road, Gulou District, Nanjing 210009, China; 5 Department of Community Health Sciences, University of Manitoba, 7750 Bannatyne Ave, Winnipeg, MB R3T 2N2, Canada; 6 NHC Key Laboratory of Food Safety Risk Assessment, China National Center for Food Safety Risk Assessment, No. 37 Guangqu Road, Chaoyang District, Beijing 100022, China

**Keywords:** DEHP, DBP, dietary exposure, extreme value mixture model, mixture model, parametric probabilistic modeling, risk assessment

## Abstract

(1) Background: Hot pot bases are susceptible to phthalate (PAE) contamination due to their high lipid content. Standard risk models often fail to capture extreme values, leading to biased exposure estimates. This study characterized dibutyl phthalate (DBP) and bis(2-ethylhexyl) phthalate (DEHP) contamination using a hybrid modeling framework to ensure precise risk profiling. (2) Methods: A total of 91 samples were analyzed via GC-MS. Concentration data were fitted using traditional parametric, extreme value mixture (EVMM), and finite mixture models. Probabilistic dietary risks were assessed for Chinese demographic groups using 10,000-iteration Monte Carlo simulations. (3) Results: DEHP (detection rate: 55%) and DBP (32%) were best modeled by a two-component Gamma mixture and a Lognormal–Generalized Pareto distribution, respectively. These advanced models significantly outperformed conventional distributions in capturing upper-tail extremes. Crucially, all hazard quotients (HQs) remained below the safety threshold of 1, indicating acceptable risk, although children aged 7–13 exhibited the highest calculated risk (Max DEHP HQ = 0.68). (4) Conclusions: Although current exposure levels are within safe limits, the heavy-tailed distributions identify potential sporadic high-exposure events that traditional models overlook, specifically highlighting the relative vulnerability of children aged 7–13. This study validates that hybrid statistical approaches offer superior precision for analyzing skewed contamination data. Consequently, these findings provide a critical scientific basis for refining regulatory monitoring and implementing targeted source-tracking measures to mitigate long-tail food safety risks.

## 1. Introduction

Phthalic acid esters (PAEs), with an annual global production of several million tons, have become pervasive environmental and food-chain contaminants [1,2,3,4]. In nationally representative U.S. biomonitoring (NHANES), recent reports indicate that urinary metabolites of dibutyl phthalate (DBP) and di(2-ethylhexyl) phthalate (DEHP) are detected in nearly all participants, indicating ubiquitous population exposure [5]. Phthalate exposure is linked to a range of adverse health effects that are primarily mediated by endocrine disruption, including interference with thyroid, estrogen, and androgen signaling [6,7]. These disturbances can lead to reproductive, developmental, and hepatic toxicity in both experimental models and humans, and epidemiological evidence also suggests a potential association between DEHP metabolites and cardiovascular disease [8,9,10,11]. Given their inherent lipophilicity and strong plastic affinity, PAEs readily migrate from plastic materials during manufacturing, storage, and transportation, leading to environmental persistence and resistance to degradation [12,13]. PAEs consequently tend to accumulate in lipid-rich food matrices, particularly in edible oils and other high-fat products [14,15]. Some types of phthalate esters, such as DEHP, which is widely used in the manufacturing and processing of plastic products (e.g., plasticizers), are already blacklisted as priority water pollutants by the U.S. Environmental Protection Agency (EPA) [16] and are included in the List of Priority Controlled Chemicals issued by the Ministry of Ecology and Environment of China [17,18,19,20,21]. Accordingly, food safety authorities have established health-based guidance values for these plasticizers; for example, the European Food Safety Authority (EFSA) has set tolerable daily intake (TDI) values of 10 µg/kg body weight/day for DBP and 50 µg/kg body weight/day for DEHP [22].

Hot pot, a traditional Chinese cuisine characterized by its distinctive flavor, has gained widespread popularity in China and internationally and has developed into a distinctive dietary culture encompassing a wide variety of regional styles [23,24]. However, contamination from production equipment and raw materials, inadequate storage and transportation conditions, insufficient regulatory oversight, and inappropriate consumer handling practices may all contribute to plasticizer contamination in hot pot soup bases. Moreover, the potential for human exposure to plasticizers from hot pot soup bases remains poorly characterized in the Chinese population, particularly among vulnerable groups such as children and adolescents. This knowledge gap underscores the urgent need for comprehensive and systematic assessments of plasticizer-related health risks in the Chinese population, with particular attention to sensitive demographic groups.

Traditional dietary risk assessment typically uses either point estimates or probabilistic models to quantify exposure, which is then compared with health-based guidance values (HBGVs) for risk characterization [25,26]. Point estimation offers a simple, deterministic approach to integrate food consumption and contaminant concentration data, but it often yields conservative exposure estimates and ignores inter-individual variability [27,28]. In contrast, probabilistic models assume that body weight, food consumption, and contaminant concentrations follow probability distributions that describe variability across individuals and food commodities [28]. These distributions may be empirical (i.e., based on representative samples) or parametric (i.e., defined by specified parameters and applicable to smaller sample sizes) [29]. Conventional distribution models (e.g., Lognormal or exponential) often fail to capture critical tail data (i.e., extreme values), potentially skewing risk assessments [30,31]. To address these constraints, this study proposes the extreme value mixture model (EVMM) and the mixture model to more accurately characterize contaminant concentration distributions in exposure assessment scenarios. The EVMM combines a distribution for the bulk of the data with a generalized Pareto distribution (GPD) for the upper tail to characterize heavy-tailed behavior. This framework enables more precise prediction of maximum contaminant concentrations and exceedance frequencies [32]. Mixture models have gained considerable attention in statistical data analysis because of their ability to represent complex distributions and play a crucial role in descriptive modeling when a single distribution is inadequate [33]. The superior performance of mixture distribution models in health-related data analysis has been demonstrated in recent studies. For example, Campolieti and Ramos compared model performance using the Akaike information criterion (AIC) and the Bayesian information criterion (BIC). Their assessment showed that mixture models consistently fit COVID-19 mortality data better than non-mixture models [34]. This finding was further supported by Fricke et al., who observed a substantial reduction in BIC values when transitioning from single-Gaussian models to more complex two-Gaussian-component additive models. Moreover, their study indicated that models incorporating three or more Gaussian components achieved greater stability and improved goodness of fit [35].

In light of these limitations, this study developed a hybrid modeling approach for probabilistic risk assessment to better capture tail distribution characteristics and improve the precision of data fitting. This approach enabled a systematic analysis of the distribution patterns of DBP and DEHP concentrations in commercially available hot pot bases in China. Furthermore, we quantitatively assessed the potential health risks associated with dietary exposure to DBP and DEHP from hot pot soup bases across different demographic groups. Therefore, we provide a scientific basis for the assessment and management of plasticizer-related health risks from hot pot soup bases in the Chinese population.

## 2. Materials and Methods

### 2.1. Chemicals and Reagents

The mixed phthalate standard solution (1000 µg/mL; purity ≥ 95%) and isotope-labeled internal standards (mixed D4-DBP and D4-DEHP, 100 μg/mL; purity 99.0% each) were obtained from BePure (Beijing, China) and stored at 0–4 °C in the dark. Detailed purities and working-solution preparation are provided in Supplementary Text S1. A certified quality-control (QC) material for phthalates in edible oil was purchased from the Testing and Evaluation Center of the Chinese Academy of Inspection and Quarantine (Beijing, China). The certified value (characteristic range) was 2.732 mg/kg (2.462–3.002 mg/kg) for DBP and 1.654 mg/kg (1.326–1.982 mg/kg) for DEHP. Primary–secondary amine (PSA) and C18 sorbents were purchased from Agilent Technologies (Santa Clara, CA, USA). Chromatographic-grade acetonitrile and n-hexane were obtained from Tianjin Kemiou Chemical Reagent Co., Ltd. (Tianjin, China). Anhydrous MgSO_4_ (analytical grade) was purchased from Chengdu Kelong Chemical Co., Ltd. (Chengdu, China).

### 2.2. Sample Collection and Preparation

A total of 91 commercially available hot pot soup base samples were purchased between September and November 2022 via both online and offline retail channels across multiple regions of China. Due to the high oil content and the presence of solid ingredients (e.g., spices) in the samples, a specific homogenization protocol was applied. Samples were frozen at −18 °C for 24 h and pulverized to ensure the uniform distribution of solid particles within the lipid matrix. The homogenized samples were then stored in stoppered glass containers at room temperature. Prior to extraction, the pulverized samples were melted in a 60 °C water bath and thoroughly shaken to ensure homogeneity. An aliquot of 0.50 g of the melted sample was weighed into a glass centrifuge tube and spiked with 40 μL of the internal standard working solution (5 μg/mL each of D4-DBP and D4-DEHP). Extraction was performed with 1 mL of n-hexane and 6 mL of acetonitrile, assisted by warm water bath incubation (10 min), vortex mixing (1 min), and ultrasonication (20 min). To facilitate the precipitation of lipid impurities, the mixture was cooled at 4 °C for 15 min before centrifugation at 3000 rpm for 5 min. The supernatant was cleaned using dispersive solid-phase extraction (d-SPE) with PSA (50 mg), C18 (50 mg), and anhydrous MgSO_4_ (100 mg). The mixture was vortexed for 1 min and centrifuged at 3000 rpm for 5 min. The cleaned extract was evaporated to dryness under a gentle nitrogen stream at 40 °C, reconstituted in 2.0 mL of n-hexane, vortexed for 1 min, and filtered through a phthalate-free 0.45 μm PTFE membrane prior to GC-MS analysis. The injection volume was 1.0 μL.

### 2.3. GC–MS Analysis

The analysis was performed on an Agilent 7890B GC (Agilent Technologies, Santa Clara, CA, USA) coupled to an Agilent 5977B MS (Agilent Technologies, Santa Clara, CA, USA) using an HP-5MS column (30 m × 0.25 mm × 0.25 μm). The MS operated in electron ionization (EI) mode (70 eV) with selected ion monitoring (SIM). Detailed instrument settings, including the oven temperature program and optimized SIM parameters, are provided in Supplementary Text S2. The compounds were identified by retention time (±0.5%) and diagnostic ion ratios using the SIM ions listed in Supplementary Table S1 and the tolerance criteria in Supplementary Table S2 (Commission Decision 2002/657/EC) [36]. Representative chromatograms are shown in Supplementary Figures S1–S3.

### 2.4. Quality Assurance and Method Performance

Strict contamination control measures were implemented. The glassware was cleaned with potassium dichromate solution, baked at 450 °C for 2 h, and rinsed with n-hexane. Reagents were pre-screened to ensure background levels were below detection limits. Quantification was performed using isotope dilution with multi-point calibration curves (0–1.00 μg/mL). Calibration solution preparation is described in Supplementary Text S1, and representative calibration curves are shown in Supplementary Figures S4 and S5. Procedural blanks were analyzed in every batch to monitor background levels; sample results were blank-corrected. Batch accuracy was verified using the certified edible-oil QC material (Certificate No. 103CO02055), with all measured values falling within the certified ranges. Precision was assessed by duplicate analyses, and accuracy was further evaluated by spike-recovery experiments at three concentration levels. The limits of detection (LOD) and quantification (LOQ) were determined in accordance with the standard GB/T 27417-2017 [37]. The LOD was estimated using a signal-to-noise (S/N) ratio of 3 in matrix-spiked samples. The LOQ was defined as 3 times the LOD. The resulting LOD/LOQ values were 0.05/0.15 mg/kg for DBP and 0.10/0.30 mg/kg for DEHP. Detailed validation data are provided in Supplementary Text S3.

### 2.5. Dietary Consumption Data Source

For the exposure assessment, consumption of hot pot base was evaluated using a dietary survey conducted among hot pot consumers in China, from 2016 to 2018 [38]. Participants were categorized into four age groups (7–13, 14–17, 18–49, and 50–78 years), and each age group was further stratified by sex to form eight subgroups. The daily consumption ranges of hot pot base and body weights for each subgroup are presented in Table 1. Consistent with the assumption that food consumption data typically follow a Lognormal distribution [39], the consumption levels were modeled using Lognormal distributions with parameters estimated from the dataset. To ensure a robust statistical representation of population patterns, 10,000 random values were generated for each demographic group based on these distributions [40].

### 2.6. Hybrid Modeling of Concentration

#### 2.6.1. Modeling of Positive Concentration Data

The positive concentration data were fitted using three modeling frameworks: a traditional parametric model, an extreme value mixture model (EVMM), and a finite mixture model. To identify the optimal model for each contaminant, a three-stage evaluation procedure was applied. In Stage 1, candidate models were screened using the Kolmogorov–Smirnov (KS) and Anderson–Darling (AD) tests, and models failing the goodness-of-fit criteria (based on *p*-values) were excluded; in Stage 2, the remaining models were ranked using AIC and BIC; in Stage 3, the top-ranked models were evaluated by 10,000 Monte Carlo simulations, with empirical cumulative distribution functions (ECDFs) and absolute deviations at the mean, P95, and P99.9, and the model with the smallest overall deviation was selected.

##### Traditional Parametric Model

Given the right-skewed concentration data, four conventional parametric distributions (Lognormal, Weibull, Gamma, and Exponential) were considered to model the positive concentration values [41].

##### Extreme Value Mixture Model

Three EVMMs were implemented: Lognormal–generalized Pareto distribution (GPD), Weibull–GPD, and Gamma–GPD. These models operate under the fundamental assumption that observations below a specified threshold (u) follow a parametric distribution H(·|η), whereas exceedances follow a generalized Pareto distribution (GPD) [42]: (Equation (1)). Parameters were estimated using the evmix (2.12) package in R (version 4.3.1) [32].

(1) $$F\left(x|\eta ,\xi ,\sigma ,u\right)=\left\{\begin{array}{cc} H(x|\eta ), & x<u\\ H(u|\eta )+[1-H(u|\eta )]G(x|\xi ,\sigma ,u), & x\geq u \end{array}\right.$$

In this equation, η represents the parameters of the bulk distribution, ξ and σ denote the shape and scale parameters of the GPD, respectively, and u indicates the location parameter. Model parameter estimation was performed using the evmix package in R, which provides specialized tools for extreme value mixture modeling.

##### Mixture Model

Three dual-distribution mixture models (2-Lognormal, 2-Weibull, and 2-Gamma) were fitted to the positive concentration data (Equation (2)). Parameters were estimated by maximum likelihood using the MixR package (0.2.0) in R (version 4.3.1) (EM algorithm), with mixing proportions constrained to sum to 1.

(2) $$f\left(x;\Phi \right)=\sum_{i=1}^{g}\;\pi_{i}f_{i}\left(x_{i};\theta_{i}\right)$$

f(x, Φ) is the probability density function (pdf) or probability mass function (pmf) of the mixture model, $f_{i}\left(x_{i};\theta_{i}\right)\;$ is the pdf or pmf of the ith component of the mixture model, $\pi_{i}$ is the proportion of the ith component and $\theta_{i}$ is the parameter of the ith component, which can be a scalar or a vector, and Φ is a vector of all the parameters of the mixture model.

#### 2.6.2. Modeling Detection Rate and Data Below the Limit of Detection

A binomial distribution, parameterized by the detection rate, was used to generate binary (0, 1) variables I. These binary variables were then multiplied by the randomly simulated positive values from the final optimal model determined in Section 2.6.1 to generate a simulated contaminant distribution of $C_{i}$.

(3) $$C_{i}=I_{i}\cdot cpos_{i}$$

where indicator variables $I_{i}$ = 1 indicate that a positive concentration was sampled, while $I_{i}$ = 0 indicates that a non-detected sample was generated. The probability of $I_{i}$ being 1 or 0 depends on the real detection rate. Values below the LOD were imputed by sampling from a uniform distribution between 0 and the LOD.

### 2.7. Exposure Estimation and Risk Characterization

Probabilistic modeling of dietary exposure was implemented by Monte Carlo (MC) simulations, which are mathematically defined as:

(4) $${EDI}_{i}\equiv \frac{{Cons}_{i}\times C_{i}}{Weight}$$

where ${Cons}_{i}$ is the daily consumption of hot pot bases by the i-th simulated individual (Section 2.5), and $C_{i}$ denotes the simulated contaminant concentration in hot pot bases consumed by the i-th person (Section 2.6.2). Weight represents the mean body weight of a certain demographic group.

The exposure estimation employed 10,000 MC iterations per demographic group. Exposure levels were evaluated at the 50th, 75th, 95th, and 99.9th percentiles, as well as the mean value of the intake distribution.

The hazard quotient (HQ) is commonly used to assess chronic exposure risk to contaminants in food. The HQ is calculated via the following formula [43]:

(5) $$HQ=\frac{EDI}{\;\mathrm{TDI}}$$

where the TDI values were recommended by the EFSA; that is, the TDI was 50 and 10 µg/(kg·bw)/day for DEHP and DBP, respectively. An HQ < 1 indicates no significant health risk, whereas an HQ ≥ 1 suggests a potential health risk.

Cumulative risk assessment for the co-exposure of DEHP and DBP was performed by calculating the Hazard Index (HI) in accordance with EFSA (2019) guidelines [44]. The Relative Potency Factor (RPF) approach was applied based on a Group-TDI of 50 µg/(kg·bw)/day (expressed as DEHP equivalents), with RPFs assigned as 1 for DEHP and 5 for DBP [44]. The HI was calculated as follows:

(6) $$\mathrm{HI}=\frac{{EDI}_{DEHP}\times 1+{EDI}_{DBP}\times 5}{\;\mathrm{Group-TDI}}$$

The uncertainty in the MC simulations of consumption and concentration estimates was evaluated using bootstrap resampling to generate approximate confidence intervals (CIs) for the specified percentiles [45]. For each demographic group, 100 bootstrap samples were generated, and the corresponding 95% confidence intervals (CIs) for both the EDI and the HQ were calculated.

### 2.8. Statistical Analysis

All statistical analyses were conducted in R (version 4.3.1), with statistical significance defined as *p* < 0.05. The analysis employed the packages stats (4.3.1), evmix (2.12), and MixR (0.2.0), whereas visualization relied on ggplot2 (3.5.1) and GridExtra (2.3).

## 3. Results

### 3.1. Description of the Consumption and Contaminant Data

To characterize the consumption distribution for each demographic group, we assumed a Lognormal distribution based on the data in Table 1 and performed a 10,000-iteration Monte Carlo simulation to generate key consumption quantiles (Table 2).

An analysis of 91 hot pot soup base samples revealed that 59% (54/91) contained detectable levels of at least one phthalate congener (DEHP or DBP). DEHP was the more prevalent contaminant, detected in 55% (50/91) of the samples, whereas DBP was detected in 32% (29/91). Table 3 presents a detailed summary for both compounds, including their mean concentrations and key percentiles among the positive samples. Co-occurrence analysis showed that 86% (25/29) of DBP-positive samples also contained DEHP, indicating a high likelihood of simultaneous exposure to multiple phthalates among consumers of hot pot soup bases. Quantitative analysis further demonstrated that DEHP concentrations (mean: 0.34 mg/kg; 99.9th percentile: 12.54 mg/kg) substantially exceeded those of DBP (mean: 0.09 mg/kg; 99.9th percentile: 0.99 mg/kg). These detection frequencies and concentration percentiles were broadly consistent with previous surveys reporting the widespread presence of lipophilic plasticizers in edible oils and other oil-rich food matrices [46].

### 3.2. Parametric Fitting of Positive Concentration Data

A hybrid modeling framework, comprising ten distinct distribution models from three categories (traditional parametric, extreme value mixture, and mixture models) was used to characterize DEHP and DBP concentrations. Because of the limited number of positive samples, DBP was excluded from the dual-component mixture model analysis and was evaluated only with traditional parametric and extreme value mixture models. The results of the goodness-of-fit tests and information criteria are summarized in Table 4. For DEHP, the KS and AD tests for the Weibull, Gamma, and Exponential models yielded *p*-values < 0.05. For DBP, the KS test for the Gamma and Exponential models also produced *p*-values < 0.05. The estimated parameters for all models are provided in Supplementary Tables S3–S5. Based on the goodness-of-fit test results and the AIC and BIC values, we selected a set of candidate models for further evaluation.

**Figure 1:**
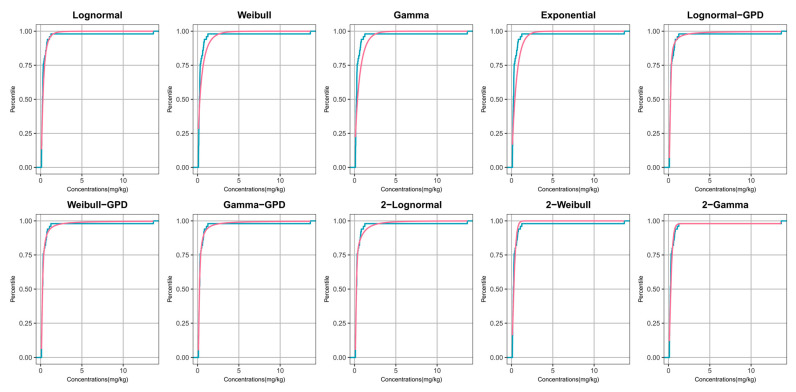
Empirical cumulative distribution plots for DEHP. Contaminant concentrations (in mg/kg) on the x-axis and percentile values on the y-axis. The actual measurements are depicted by a teal curve—each vertex representing a specific data point—while the theoretical fits are shown in pink.

**Figure 2:**
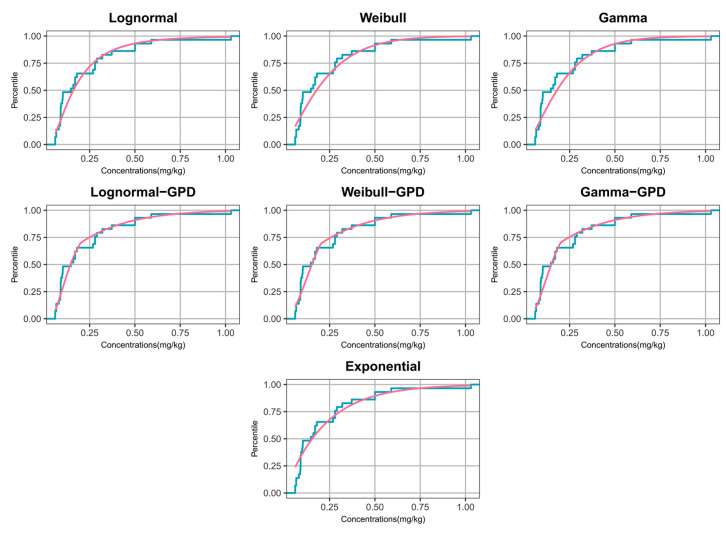
Empirical cumulative distribution plots for DBP. Contaminant concentrations (in mg/kg) on the x-axis and percentile values on the y-axis. The actual measurements are depicted by a teal curve—each vertex representing a specific data point—while the theoretical fits are shown in pink.

Model performance was evaluated by overlaying the empirical CDF (step function) with the fitted CDF (smooth curve) from each candidate model (Figure 1 and Figure 2), with particular attention to both the bulk region and the upper tail. For DEHP, several single-distribution models that were rejected by KS/AD tests (e.g., Weibull, Gamma, and Exponential) showed visible departures from the ECDF, whereas the 2-Gamma, Lognormal–GPD, and Lognormal models provided the closest overall agreement (Figure 1). Notably, the Lognormal and Lognormal–GPD model tended to under-represent extreme upper-tail probabilities; the 2-Gamma model achieved a better balance between bulk fit and tail representation. For DBP, the Lognormal–GPD model tracked the ECDF more closely in the upper tail than single-distribution models, whereas some traditional parametric models showed systematic deviations consistent with the goodness-of-fit results (Figure 2). Based on visual assessment and statistical criteria (non-significant goodness-of-fit tests and the smallest BIC and AIC values), the candidate models selected for further evaluation were the 2-Gamma, Lognormal-GPD, and Lognormal models for DEHP, and the Lognormal–GPD and Lognormal models for DBP.

Subsequently, Monte Carlo simulations (*n* = 10,000) were performed to generate selected percentiles from these models, which were then compared with the corresponding empirical percentiles (Table 5 and Table 6). This percentile-based comparison complements the ECDF diagnostics by quantifying model performance at decision-relevant points, particularly in the extreme upper tail (P99.9), which is critical for conservative risk characterization. For DEHP (Table 5), the 2-Gamma model showed the closest agreement between simulated and observed values, with relative differences of 7.59% for the mean, 11.82% for the 95th percentile, and 4.66% for the 99.9th percentile. For DBP (Table 6), although the Lognormal–GPD model showed a slightly poorer fit at the 95th percentile (relative difference of 12.02% from the measured value), it outperformed the Lognormal model across all other evaluated percentiles. Adopting a conservative approach that prioritizes performance at the 99.9th percentile, we identified the 2-Gamma model as the optimal fit for DEHP and the Lognormal–GPD model as the optimal fit for DBP.

### 3.3. Dietary Exposure Levels and Risk Assessment

Concentration data simulated from the optimal models were integrated with consumption data to estimate the distributions of EDI and the corresponding HQ for each contaminant using Equations (4) and (5). The 95% CIs for selected exposure percentiles were estimated using 100 bootstrap iterations. Table 7 presents the detailed distributions of EDIs, along with the HQs at mean and 95th percentile (P95) levels to highlight key risk scenarios. Complete data regarding HQs and their 95% CIs are provided in Table S6.

Regarding dietary exposure, Table 7 shows that children aged 7–13 years exhibited the highest intake levels per body weight for both contaminants. In terms of risk characterization, across all demographic groups, no significant non-carcinogenic health risks were identified, as all HQs remained below the safety threshold of 1. As shown in Table 7, HQs at mean exposure levels were approximately 0.010 for both compounds, indicating negligible risk at central tendencies, while higher values were observed at the 95th percentile and above.

Extreme exposure scenarios were further analyzed. As detailed in Table 7, the estimated intakes at the 99.9th percentile were notably high, particularly for females aged 7–13 years, reaching 34.025 µg/(kg·bw)/day for DEHP and 2.232 µg/(kg·bw)/day for DBP. The highest HQ for DEHP exposure at the 99.9th percentile was observed among females aged 7–13 years (HQ = 0.681; 95% CI, 0.571–0.767) (Table S6), which exceeded those in all other demographic groups. For DBP exposure, the 99.9th percentile HQ ranged from 0.15 to 0.25 (Table S6), with the highest values again observed in children aged 7–13 years. In magnitude, these exposure estimates were broadly comparable to those reported for edible-oil–related assessments in western China [47] but lower than whole-diet-based estimates reported in population-specific studies [48].

**Figure 3:**
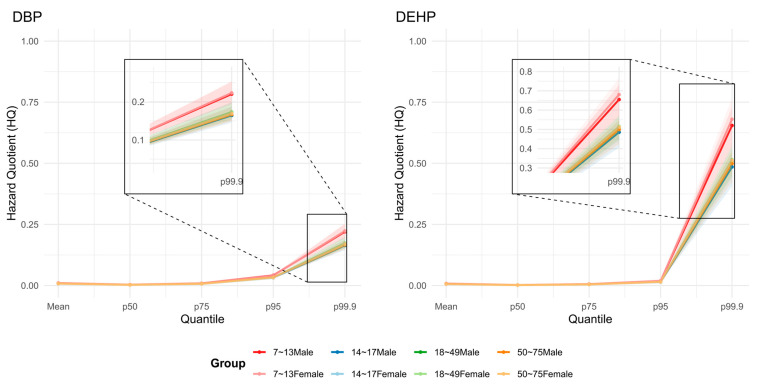
The gender–age stratified HQ values with uncertainty intervals for DEHP and DBP exposure from hot pot bases in the Chinese population.

Figure 3 displays the quantile distribution of HQ for each demographic group, with HQ values plotted on the vertical axis against their corresponding quantiles on the horizontal axis. This figure illustrates that for both contaminants, the 7–13-year-old groups (males in red, females in pink) exhibit the highest HQ values, particularly at the upper quantiles (e.g., the 99.9th percentile). The 95% CIs indicated that the mean and lower exposure percentiles were relatively stable, whereas the higher percentiles showed substantially greater uncertainty, reflecting larger sampling variability in the upper tail.

Under the cumulative risk assessment framework, the highest HI was observed in females aged 7–13 at the extreme 99.9th percentile, reaching a value of 0.904 (Table 8). Crucially, even this worst-case estimate remained below the safety threshold of 1.

## 4. Discussion

This study combined a hybrid distributional modeling framework with a probabilistic exposure assessment to characterize dietary exposure to DEHP and DBP from hot pot soup bases. A substantial proportion of samples (59%) contained detectable levels of at least one phthalate. DEHP was detected more frequently (55%) than DBP (32%), and its concentrations were consistently higher. The frequent co-occurrence of DEHP and DBP in positive samples (86%) suggests that consumers are likely to experience simultaneous exposure to multiple phthalates through this product category. Such patterns are consistent with surveys of edible oils and other fat-rich foods, in which DBP and DEHP are repeatedly reported as dominant congeners, supporting the propensity of oil-based matrices to accumulate these plasticizers. For example, an investigation of imported plant oils in western China reported that PAEs (mainly DBP and DEHP) were detected in up to 56.83% of 366 imported plant oil samples [47]. Across food-monitoring datasets, cooking oils have been highlighted as a food group with relatively high DEHP concentrations, reinforcing the relevance of oil-based foods as an important dietary source of DEHP [49]. This matrix dependence is further supported by migration evidence showing that packaged cooking oil facilitates higher PAE migration from plastic containers than mineral water under comparable conditions [50].

In terms of exposure magnitude, our findings indicate that phthalate exposure levels from hot pot soup bases are broadly comparable to those reported for imported vegetable oils in western China, as assessed by Tang et al. [47]. By contrast, our estimated dietary exposure levels of DEHP and DBP are substantially lower than those reported for pregnant women by Shan et al. [48] and lower than the estimated maximum daily intake of DEHP and DBP from edible oils reported by Luo and colleagues [51]. This discrepancy is anticipated given that previous studies typically assessed total dietary intake (or multiple major food sources) rather than a single product category. Additionally, variations in target populations and exposure-scenario definitions may further contribute to the observed between-study variability. Thus, the lower absolute exposure levels observed in our study should be interpreted within the narrower context of hot pot soup base consumption rather than overall dietary intake.

Although all estimated HQs in our study remained below 1, indicating no apparent non-carcinogenic health concern under current exposure conditions, the upper-tail estimates warrant attention. At the 99.9th percentile, DEHP HQs were considerably higher than those for DBP, with the highest values observed among children aged 7–13 years. This age group consistently exhibited the greatest risk across both contaminants, reflecting higher intake relative to body weight. The finding that DEHP posed a relatively higher risk than DBP at extreme percentiles is consistent with previous work that has identified DEHP as a major contributor to overall phthalate exposure [47]. Furthermore, the biomonitoring study by Jeddi et al. [52] in Iranian children and adolescents reported combined exposure dominated by DEHP and DBP, and Dewalque et al. [53] highlighted that children are more vulnerable to the high-risk impacts of PAEs than adults are. These external data align with our observation that school-aged children represent a key at-risk group for high-end exposure, even when average exposure levels appear acceptable.

Our results are also consistent with previous dietary risk assessments indicating that mean or median PAE exposure typically falls below health-based guidance values but that upper-bound estimates can approach or exceed TDI thresholds. For example, Gkrillas et al. [54] reported that PAE exposure from food remained below the daily tolerable intake levels established by the EFSA, but could approach approximately twice the TDI under upper-bound scenarios. Similarly, in our assessment, central-tendency HQs were close to 0.01 for both compounds, whereas upper-tail HQs for DEHP approached levels that merit closer scrutiny in high-consumption subgroups. Taken together, these findings support the view that, while typical dietary exposure to PAEs from individual food categories may not pose immediate non-carcinogenic concerns, high-end exposure scenarios—particularly among children—require continued monitoring and periodic re-evaluation.

The probabilistic framework used here also highlights the importance of explicitly characterizing uncertainty in upper-tail risk estimates. The 95% CIs around mean and lower-percentile HQs were narrow, indicating stable estimates. In contrast, CIs at the 95th and 99.9th percentiles were considerably wider, reflecting the influence of sparse extreme concentration values on model fitting and simulation. This pattern is expected in the context of limited sample sizes and rare extreme events, but it underscores the need to interpret high-percentile estimates with caution and to report uncertainty intervals alongside point estimates in dietary risk assessments.

Several limitations of this study should be acknowledged. First, the relatively small sample size contributed to high variability in the observed concentration data, particularly for DEHP, thereby introducing additional uncertainty into the parameter estimates and model performance. While the Lognormal–GPD model was robust for predicting extreme values (P99.9) for DBP, it was less accurate at the P95. Nonetheless, this model was retained because it provided conservative estimates in the upper tail, which is of primary interest for health protection. Second, although uncertainty was quantified using bootstrapping, the resulting 95% CIs at the upper percentiles remained wide, which is inherent to tail-end estimation based on limited data. Third, consumption was characterized from survey-based intake ranges rather than individual-level dietary records, which may introduce additional exposure misclassification. Finally, this assessment focused on oral exposure to two target phthalates from a single food category and did not account for cumulative exposure to multiple phthalates or for other routes such as dermal contact and inhalation. Finally, due to data constraints, the HI was calculated using only DEHP and DBP. Since the EFSA Group-TDI also includes BBP and DINP, this partial assessment may result in a slight underestimation of the cumulative risk.

Despite these limitations, the present work demonstrates that extreme value and mixture models can provide more precise and informative estimates for probabilistic dietary risk assessment than traditional single-distribution approaches, particularly when contaminant data include extreme but plausible values. To better characterize phthalate exposure from hot pot soup bases, future studies should increase both the sample size and sampling representativeness by including products from a wider range of brands, geographic regions, and production seasons. This expanded sampling strategy will help clarify whether the observed extreme concentrations represent sporadic events or indicate systemic contamination patterns. In addition, targeted source-tracking investigations should be conducted for samples exhibiting extreme concentrations, with particular attention to raw material sourcing, processing conditions, and migration from packaging materials. Such efforts will facilitate the identification of critical contamination sources and support the development of targeted mitigation strategies, ultimately contributing to more effective risk management for phthalate exposure in the general population, especially among vulnerable groups such as children and pregnant women.

## 5. Conclusions

This study systematically analyzed the concentrations of DEHP and DBP in hot pot bases and conducted a probabilistic risk assessment. Using a hybrid statistical modeling approach, DEHP was best described by a two-component Gamma (2-Gamma) distribution, whereas DBP was best described by a Lognormal–Generalized Pareto (Lognormal–GPD) distribution. These models effectively predicted both the mean concentrations and the 99.9th percentile (P99.9) levels.

Overall, the estimated chronic risks for the evaluated demographic groups were generally low under the current exposure assumptions (all HQs < 1). However, the modeled upper tail (P99.9) suggests potential “long-tail” risks driven by sporadic high-contamination events, with children aged 7–13 years representing the most sensitive subgroup. This underscores the need for continued monitoring and further source tracing, as well as methodological improvements to reduce uncertainty and better distinguish sporadic incidents from systematic pollution. These findings provide a scientific basis for prioritizing targeted monitoring and risk-management actions.

## Figures and Tables

**Table 1:** Consumption and demographic information of the consumers of hot pot bases in China.

Groups	Age (Years)	Gender	Consumption (g/d)	Average Weight (kg)
M, 7–13 years	7–13	male	10.0–120.0	39.30
F, 7–13 years	7–13	female	6.7–116.7	36.35
M, 14–17 years	14–17	male	13.3–133.3	59.75
F, 14–17 years	14–17	female	8.3–125.0	51.7
M, 18–49 years	18–49	male	15.0–158.3	67.35
F, 18–49 years	18–49	female	11.7–133.1	56.15
M, 50–75 years	50–75	male	16.7–141.7	63.15
F, 50–75 years	50–75	female	11.7–125.0	53.45

Consumption: the daily consumption range of hot pot base for this group; M: male; F: female. Age groups are denoted as [lower bound]–[upper bound] years.

**Table 2:** Simulated daily consumption of the consumers of hot pot bases in China (g/d).

Groups	Consumption (g/d)				
	Mean	P50	P75	P95	P99.9
M, 7–13 years	45.9	37.2	66.1	106.8	120.0
F, 7–13 years	41.1	31.7	60.2	102.6	109.5
M, 14–17 years	53.2	44.3	75.9	119.1	126.3
F, 14–17 years	45.3	35.5	66.2	110.3	117.6
M, 18–49 years	62.1	51.3	88.8	140.6	149.5
F, 18–49 years	51.5	42.1	74.0	118.6	125.7
M, 50–75 years	59.2	50.2	83.3	127.3	134.8
F, 50–75 years	49.2	40.7	70.2	111.8	118.4

P: percentile; M: male; F: female. Age groups were denoted as [lower bound]–[upper bound] years.

**Table 3:** Detection rates and the positive concentration level of DEHP and DBP in the collected samples in the year 2022.

Contaminants	Sample Size	Detection Rate (%)	Concentration (mg/kg)				
			Mean	P50	P75	P95	P99.9
DBP	91	32	0.09	0.04	0.09	0.35	0.99
DEHP	91	55	0.34	0.11	0.26	0.74	12.54

**Table 4:** Summary on fitting of the distribution for the concentrations of DEHP and DBP in the hot pot bases.

Distribution	DEHP				DBP			
	KS	AD	AIC	BIC	KS	AD	AIC	BIC
Traditional parametric models								
Lognormal	0.08	0.11	−1.87	1.96	0.17	0.42	−35.54	−32.81
Weibull	<0.01	<0.01	37.42	41.24	0.26	0.26	−27.53	−24.79
Gamma	<0.01	<0.01	47.48	51.30	0.03	0.25	−29.51	−26.77
Exponential	<0.01	<0.01	47.75	49.66	<0.01	0.18	−27.36	−25.99
EVMM								
Lognormal-GPD	0.92	0.67	−17.41	−9.76	0.23	0.54	−38.45	−35.71
Weibull-GPD	0.58	0.35	−8.36	−0.71	0.18	0.44	−33.81	−31.07
Gamma-GPD	0.84	0.47	−13.09	−5.44	0.16	0.45	−35.88	−33.14
Mixture								
2-Lognormal	0.73	0.75	−15.63	−6.07	NA	NA	NA	NA
2-Weibull	0.06	0.08	−8.62	0.94	NA	NA	NA	NA
2-Gamma	0.09	0.11	−35.76	−26.20	NA	NA	NA	NA

KS: *p* value of the Kolmogorov—Smirnov test; AD: *p* value of the Anderson—Darling test; AIC: Akaike information criterion; BIC: Bayesian information criterion; EVMM: extreme value mixture model; mixture: mixture model; NA: not available.

**Table 5:** Relative differences on the percentiles of DEHP concentrations between simulated values and original detection.

Parameter	Original Value (mg/kg)	Model	Estimated Value (mg/kg)	Relative Difference from Measured Value (%)
Mean	0.5813	Lognormal–GPD	0.5332	8.27
		Lognormal	0.3847	33.82
		2-Gamma	0.6254	7.59
P50	0.2360	Lognormal–GPD	0.2368	0.34
		Lognormal	0.2671	13.18
		2-Gamma	0.2756	16.78
P90	0.7317	Lognormal–GPD	0.6581	10.06
		Lognormal	0.8026	9.69
		2-Gamma	0.6526	10.81
P95	0.9572	Lognormal–GPD	1.1361	18.69
		Lognormal	1.0807	12.90
		2-Gamma	0.8441	11.82
P99.9	13.0517	Lognormal–GPD	31.3602	140.28
		Lognormal	3.7124	71.56
		2-Gamma	13.6600	4.66

**Table 6:** Relative differences on the percentiles of DBP concentrations between simulated values and original detection.

Parameter	Original Value (mg/kg)	Model	Estimated Value (mg/kg)	Relative Difference from Measured Value (%)
Mean	0.2218	Lognormal–GPD	0.2204	0.63
		Lognormal	0.2106	5.05
P50	0.1465	Lognormal–GPD	0.1434	2.12
		Lognormal	0.1650	12.63
P90	0.5000	Lognormal–GPD	0.4930	1.40
		Lognormal	0.4280	14.40
P95	0.5540	Lognormal–GPD	0.6206	12.02
		Lognormal	0.5920	6.86
P99.9	1.0177	Lognormal–GPD	1.3922	36.80
		Lognormal	1.7137	68.39

**Table 7:** EDIs and corresponding HQs for exposure to DEHP and DBP from hot pot bases in the Chinese population.

Contaminants	Group	EDI (95%CI) µg/(kg·bw)/day					HQs	
		Mean	P50	P75	P95	P99.9	Mean	P95
DEHP	M, 7–13 years	0.400 (0.360, 0.435)	0.110 (0.105, 0.115)	0.280 (0.270, 0.290)	0.975 (0.930, 1.015)	32.740 (27.830, 36.560)	0.008	0.020
	F, 7–13 years	0.385 (0.355, 0.420)	0.100 (0.095, 0.105)	0.260 (0.250, 0.270)	0.980 (0.935, 1.025)	34.025 (28.530, 38.350)	0.008	0.020
	M, 14–17 years	0.305 (0.270, 0.335)	0.085 (0.085, 0.090)	0.220 (0.210, 0.225)	0.730 (0.695, 0.765)	24.270 (20.305, 27.350)	0.006	0.015
	F, 14–17 years	0.300 (0.270, 0.330)	0.080 (0.075, 0.080)	0.205 (0.200, 0.215)	0.750 (0.720, 0.790)	25.420 (21.570, 28.730)	0.006	0.015
	M, 18–49 years	0.320 (0.290, 0.345)	0.090 (0.085, 0.090)	0.225 (0.220, 0.235)	0.760 (0.730, 0.790)	25.515 (22.305, 28.225)	0.006	0.015
	F, 18–49 years	0.315 (0.280, 0.345)	0.085 (0.085, 0.090)	0.225 (0.215, 0.230)	0.760 (0.730, 0.800)	25.755 (22.525, 29.085)	0.006	0.015
	M, 50–75 years	0.325 (0.295, 0.350)	0.095 (0.090, 0.095)	0.235 (0.225, 0.245)	0.755 (0.725, 0.785)	24.955 (21.165, 27.630)	0.007	0.015
	F, 50–75 years	0.315 (0.290, 0.345)	0.090 (0.085, 0.090)	0.225 (0.220, 0.235)	0.760 (0.725, 0.800)	25.580 (23.125, 27.705)	0.006	0.015
DBP	M, 7–13 years	0.100 (0.096, 0.104)	0.035 (0.034, 0.036)	0.089 (0.086, 0.092)	0.416 (0.392, 0.439)	2.201 (1.933, 2.524)	0.010	0.042
	F, 7–13 years	0.096 (0.092, 0.101)	0.032 (0.031, 0.033)	0.085 (0.082, 0.088)	0.404 (0.380, 0.426)	2.232 (1.947, 2.537)	0.010	0.040
	M, 14–17 years	0.077 (0.074, 0.080)	0.027 (0.026, 0.028)	0.069 (0.066, 0.071)	0.318 (0.301, 0.336)	1.642 (1.455, 1.876)	0.008	0.032
	F, 14–17 years	0.075 (0.072, 0.078)	0.025 (0.024, 0.026)	0.066 (0.064, 0.069)	0.314 (0.297, 0.331)	1.721 (1.505, 1.980)	0.008	0.031
	M, 18–49 years	0.080 (0.076, 0.082)	0.028 (0.027, 0.029)	0.071 (0.069, 0.073)	0.328 (0.312, 0.347)	1.728 (1.538, 1.993)	0.008	0.033
	F, 18–49 years	0.079 (0.076, 0.081)	0.028 (0.026, 0.028)	0.070 (0.068, 0.072)	0.327 (0.310, 0.341)	1.722 (1.530, 1.944)	0.008	0.033
	M, 50–75 years	0.081 (0.077, 0.084)	0.029 (0.028, 0.030)	0.073 (0.071, 0.075)	0.332 (0.313, 0.353)	1.665 (1.488, 1.858)	0.008	0.033
	F, 50–75 years	0.079 (0.076, 0.082)	0.028 (0.027, 0.029)	0.071 (0.068, 0.073)	0.327(0.309, 0.347)	1.691 (1.508, 1.931)	0.008	0.033

**Table 8:** Estimated HIs for Chinese demographic groups under cumulative exposure scenarios.

Group	HIs				
	Mean	P50	P75	P95	P99.9
M, 7–13 years	0.018	0.006	0.015	0.061	0.875
F, 7–13 years	0.017	0.005	0.014	0.060	0.904
M, 14–17 years	0.014	0.004	0.011	0.046	0.650
F, 14–17 years	0.014	0.004	0.011	0.046	0.681
M, 18–49 years	0.014	0.005	0.012	0.048	0.683
F, 18–49 years	0.014	0.005	0.012	0.048	0.687
M, 50–75 years	0.015	0.005	0.012	0.048	0.666
F, 50–75 years	0.014	0.005	0.012	0.048	0.681

## Data Availability

The data presented in this study are available on request from the corresponding author.

## Supplementary Materials

The following supporting information can be downloaded at: https://www.mdpi.com/article/10.3390/toxics14020150/s1, Supplementary Text S1: Standard Solutions and Calibration; Supplementary Text S2: GC-MS Parameters; Supplementary Text S3: Method validation results; Figure S1: Total ion chromatogram (TIC) of a procedural blank processed using the QuEChERS–GC–MS method; Figure S2: Total ion chromatogram (TIC) of the mixed PAE standard solution under the optimized GC–MS conditions; Figure S3: Representative total ion chromatogram (TIC) of a hot pot soup base sample under the optimized GC–MS conditions; Figure S4: Calibration curve for DBP obtained by isotope-dilution GC–MS (multi-point external calibration with internal-standard correction); Figure S5: Calibration curve for DEHP obtained by isotope-dilution GC–MS (multi-point external calibration with internal-standard correction); Table S1: Retention times and monitoring ions for target compounds and internal standards; Table S2: Maximum permitted tolerances for relative ion intensities in GC-MS qualitative confirmation (Reference: Commission Decision 2002/657/EC); Table S3: The fitting parameters of the positive concentration of the traditional parametric model for DEHP and DBP; Table S4: The fitting parameters of the positive concentration of the extreme value mixture model for DEHP and DBP; Table S5: The fitting parameters of the positive concentration of the mixture model for DEHP; Table S6: Hazard quotients (HQs) and 95% confidence intervals (CIs) for exposure to DEHP and DBP from hot pot bases in the Chinese population.

## Abbreviations

The following abbreviations are used in this manuscript:

AD	Anderson–Darling (test)
AIC	Akaike information criterion
BIC	Bayesian information criterion
CI	Confidence interval
CVD	Cardiovascular disease
DBP	Dibutyl phthalate
DEHP	Bis(2-ethylhexyl) phthalate
d-SPE	Dispersive Solid Phase Extraction
ECDF	Empirical cumulative distribution function
EDI	Estimated daily intake
EFSA	European Food Safety Authority
EI	Electron ionization
EPA	Environmental Protection Agency
EVMM	Extreme value mixture model
GC-MS	Gas Chromatography–Mass Spectrometry
GPD	Generalized Pareto distribution
HBGV	Health-based guidance value
HQ	Hazard quotient
KS	Kolmogorov–Smirnov (test)
LOD	Limit of detection
MC	Monte Carlo (simulation)
PAEs	Phthalic acid esters
PSA	Primary-Secondary Amine
QA/QC	Quality Assurance and Quality Control
RSD	Relative Standard Deviation
SIM	Selected ion monitoring
TDI	Tolerable daily intake

## References

[1] Nanni N., Fiselier K., Grob K., Di Pasquale M., Fabrizi L., Aureli P., Coni E. (2011). Contamination of vegetable oils marketed in Italy by phthalic acid esters. Food Control.

[2] OECD (2018). Considerations for Assessing the Risks of Combined Exposure to Multiple Chemicals.

[3] Lazăr N.N., Călmuc M., Milea Ș.A., Georgescu P.L., Iticescu C. (2024). Micro and nano plastics in fruits and vegetables: A review. Heliyon.

[4] Giuliani A., Zuccarini M., Cichelli A., Khan H., Reale M. (2020). Critical Review on the Presence of Phthalates in Food and Evidence of Their Biological Impact. Int. J. Environ. Res. Public Health.

[5] National Center for Environmental Health (U.S.), Division of Laboratory Sciences (2019). Fourth National Report on Human Exposure to Environmental Chemicals: Updated Tables, January 2019, Volume One.

[6] Yang T.C., Jovanovic N., Chong F., Worcester M., Sakhi A.K., Thomsen C., Garlantézec R., Chevrier C., Jensen G., Cingotti N. (2023). Interventions to Reduce Exposure to Synthetic Phenols and Phthalates from Dietary Intake and Personal Care Products: A Scoping Review. Curr. Environ. Health Rep..

[7] Kahn L.G., Philippat C., Nakayama S.F., Slama R., Trasande L. (2020). Endocrine-disrupting chemicals: Implications for human health. Lancet Diabetes Endocrinol..

[8] Gray L.E., Ostby J., Furr J., Price M., Veeramachaneni D.N., Parks L. (2000). Perinatal exposure to the phthalates DEHP, BBP, and DINP, but not DEP, DMP, or DOTP, alters sexual differentiation of the male rat. Toxicol. Sci..

[9] Lyche J.L., Gutleb A.C., Bergman A., Eriksen G.S., Murk A.J., Ropstad E., Saunders M., Skaare J.U. (2009). Reproductive and developmental toxicity of phthalates. J. Toxicol. Environ. Health B Crit. Rev..

[10] Chen X., Xu S., Tan T., Lee S.T., Cheng S.H., Lee F.W., Xu S.J., Ho K.C. (2014). Toxicity and estrogenic endocrine disrupting activity of phthalates and their mixtures. Int. J. Environ. Res. Public Health.

[11] Wen Z.J., Wang Z.Y., Zhang Y.F. (2022). Adverse cardiovascular effects and potential molecular mechanisms of DEHP and its metabolites—A review. Sci. Total Environ..

[12] Nie D., Dai F. (2023). Pollution level and exposure assessment of three main phthalates in commercial vegetable oils in Weifang City. Chin. J. Health Lab. Technol..

[13] Sokołowski A., Kończak M., Oleszczuk P., Gao Y., Czech B. (2024). Environmental and food contamination by phthalic acid esters (PAEs): Overview. Water Air Soil Pollut..

[14] Moskovkin A. (2002). Chromatographic–mass-spectrometric determination of toxic substances liberated from polymeric materials. J. Anal. Chem..

[15] Lacoste F. (2014). Undesirable substances in vegetable oils: Anything to declare?. OCL.

[16] U.S. Environmental Protection Agency (2014). Appendix A to Part 423—126 Priority Pollutants.

[17] Planelló R., Herrero O., Martínez-Guitarte J.L., Morcillo G. (2011). Comparative effects of butyl benzyl phthalate (BBP) and di(2-ethylhexyl) phthalate (DEHP) on the aquatic larvae of Chironomus riparius based on gene expression assays related to the endocrine system, the stress response and ribosomes. Aquat. Toxicol..

[18] Gao D.W., Wen Z.D. (2016). Phthalate esters in the environment: A critical review of their occurrence, biodegradation, and removal during wastewater treatment processes. Sci. Total Environ..

[19] European Commission (2007). Commission Staff Working Document on the Implementation for the “Community Strategy for Endocrine Disrupters”—A Range of Substances Suspected of Interfering with the Hormone Systems of Humans and Wildlife (COM (1999) 706), (COM (2001) 262) and (SEC (2004) 1372).

[20] Net S., Sempéré R., Delmont A., Paluselli A., Ouddane B. (2015). Occurrence, fate, behavior and ecotoxicological state of phthalates in different environmental matrices. Environ. Sci. Technol..

[21] Ministry of Ecology and Environment of the People’s Republic of China (2017). The List of Priority Controlled Chemicals (First Batch).

[22] EFSA (2005). Opinion of the Scientific Panel on Food Additives, Flavourings, Processing Aids and Materials in Contact with Food (AFC) on a request from the Commission related to Butylbenzylphthalate (BBzP) for use in food contact materials. EFSA J..

[23] Yang L., Zhang M., Jia H.-F., Tu M.-J., Huang Y., Song L.-S., Yan L.-Q. (2021). Modelling for Pungency Grading of Spicy Hot Pot Seasonings Based on Capsaicinoid Content Determined by HPLC and Analysis of Its Changes During Boiling. Food Sci..

[24] Wu M., Guo P., Tsui S.W., Chen H., Zhao Z. (2012). An ethnobotanical survey of medicinal spices used in Chinese hotpot. Food Res. Int..

[25] Hamilton D., Ambrus A., Dieterle R., Felsot A., Harris C., Petersen B., Racke K., Wong S.S., Gonzalez R., Tanaka K. (2004). Pesticide residues in food—Acute dietary exposure. Pest Manag. Sci..

[26] Simionov I.A., Călmuc M., Iticescu C., Călmuc V., Georgescu P.L., Faggio C., Petrea Ş.M. (2023). Human health risk assessment of potentially toxic elements and microplastics accumulation in products from the Danube River Basin fish market. Environ. Toxicol. Pharmacol..

[27] Finley B., Paustenbach D. (1994). The benefits of probabilistic exposure assessment: Three case studies involving contaminated air, water, and soil. Risk Anal..

[28] Paulo M.J., van der Voet H., Jansen M.J., ter Braak C.J., van Klaveren J.D. (2005). Risk assessment of dietary exposure to pesticides using a Bayesian method. Pest Manag. Sci..

[29] de Boer W.J., van der Voet H. (2006). MCRA, Release 5, a Web-Based Program for Monte Carlo Risk Assessment.

[30] Bertail P., Tressou J. (2006). Incomplete generalized U-statistics for food risk assessment. Biometrics.

[31] Gauchi J.P., Leblanc J.C. (2002). Quantitative assessment of exposure to the mycotoxin ochratoxin A in food. Risk Anal..

[32] Hu Y., Scarrott C. (2018). evmix: An R package for extreme value mixture modeling, threshold estimation and boundary corrected kernel density estimation. J. Stat. Softw..

[33] McLachlan G.J., Jones P.N. (1988). Fitting mixture models to grouped and truncated data via the EM algorithm. Biometrics.

[34] Campolieti M., Ramos A. (2022). The distribution of COVID-19 mortality. Infect. Dis. Model..

[35] Fricke T.R., Keay L., Resnikoff S., Tahhan N., Koumbo O., Paudel P., Ayton L.N., Britten-Jones A.C., Kweon S., Li J.C.H. (2023). Improving population-level refractive error monitoring via mixture distributions. Ophthalmic Physiol. Opt..

[36] European Commission (2002). Commission Decision 2002/657/EC implementing Council Directive 96/23/EC concerning the performance of analytical methods and the interpretation of results. Off. J. Eur. Commun..

[37] (2017). Conformity Assessment—Guidance on Validation and Verification of Chemical Analytical Methods.

[38] Lianji Z. (2020). Comprehensive Evaluation on the Safety of Chongqing Spicy Hotpot Seasoning and the Mechanism of Intestinal Intervention with Compound Powder. Ph.D. Thesis.

[39] Slob W. (2006). Probabilistic dietary exposure assessment taking into account variability in both amount and frequency of consumption. Food Chem. Toxicol..

[40] Yue H., Wei L.W., Bo C., Jie Y., Min W., Guang L.S. (2014). Phthalates in Commercial Chinese Rice Wines: Concentrations and the Cumulative Risk Assessment to Adult Males in Shanghai. Biomed. Environ. Sci..

[41] Li J. (2010). Study on Parametric Probabilistic Exposure Assessment Model in Dietary Cypermethrin. Master’s Thesis.

[42] Koch H., Starenki D., Cooper S.J., Myers R.M., Li Q. (2018). powerTCR: A model-based approach to comparative analysis of the clone size distribution of the T cell receptor repertoire. PLoS Comput. Biol..

[43] NRC (2008). Phthalates and Cumulative Risk Assessment: The Tasks Ahead.

[44] Silano V., Barat Baviera J.M., Bolognesi C., Chesson A., Cocconcelli P.S., Crebelli R., Gott D.M., Grob K., Lampi E., Mortensen A. (2019). Update of the risk assessment of di-butylphthalate (DBP), butyl-benzyl-phthalate (BBP), bis(2-ethylhexyl)phthalate (DEHP), di-isononylphthalate (DINP) and di-isodecylphthalate (DIDP) for use in food contact materials. EFSA J..

[45] Kettler S., Kennedy M., McNamara C., Oberdörfer R., O’Mahony C., Schnabel J., Smith B., Sprong C., Faludi R., Tennant D. (2015). Assessing and reporting uncertainties in dietary exposure analysis: Mapping of uncertainties in a tiered approach. Food Chem. Toxicol..

[46] Kıralan S.S., Toptancı İ., Öncül Abacıgil T., Ramadan M.F. (2020). Phthalates levels in olive oils and olive pomace oils marketed in Turkey. Food Addit. Contam. Part A Chem. Anal. Control Expo. Risk Assess..

[47] Tang Z., Gong Z., Jia W., Shen W., Han Q., Fang F., Peng C. (2022). Occurrence and exposure risk assessment of phthalate esters in edible plant oils with a high-frequency import rate in west China. RSC Adv..

[48] Shan D., Zhang T., Li L., Sun Y., Wang D., Li Y., Yang Z., Cui K., Wu S., Jin L. (2022). Cumulative risk assessment of dietary exposure to phthalates in pregnant women in Beijing, China. Environ. Sci. Pollut. Res. Int..

[49] Serrano S.E., Braun J., Trasande L., Dills R., Sathyanarayana S. (2014). Phthalates and diet: A review of the food monitoring and epidemiology data. Environ. Health.

[50] Xu Q., Yin X., Wang M., Wang H., Zhang N., Shen Y., Xu S., Zhang L., Gu Z. (2010). Analysis of phthalate migration from plastic containers to packaged cooking oil and mineral water. J. Agric. Food Chem..

[51] Luo Q., Liu Z.H., Yin H., Dang Z., Wu P.X., Zhu N.W., Lin Z., Liu Y. (2020). Global review of phthalates in edible oil: An emerging and nonnegligible exposure source to human. Sci. Total Environ..

[52] Zare Jeddi M., Eshaghi Gorji M., Rietjens I., Louisse J., Bruinen de Bruin Y., Liska R. (2018). Biomonitoring and Subsequent Risk Assessment of Combined Exposure to Phthalates in Iranian Children and Adolescents. Int. J. Environ. Res. Public Health.

[53] Dewalque L., Charlier C., Pirard C. (2014). Estimated daily intake and cumulative risk assessment of phthalate diesters in a Belgian general population. Toxicol. Lett..

[54] Gkrillas A., Dirven H., Papadopoulou E., Andreassen M., Hjertholm H., Husøy T. (2021). Exposure estimates of phthalates and DINCH from foods and personal care products in comparison with biomonitoring data in 24-hour urine from the Norwegian EuroMix biomonitoring study. Environ. Int..

