# Supplementary material for: Risk Assessment of Dibutyl Phthalate (DBP) and Bis(2-Ethylhexyl) Phthalate (DEHP) in Hot Pot Bases with a Hybrid Modeling Approach
Source: Toxics. 2026 Feb 2;14(2):150. doi: 10.3390/toxics14020150 (PMC12945265; doi:10.3390/toxics14020150)
Supplement: Supplementary file 1 [file toxics-14-00150-s001.zip › toxics-4134334-supplementary.pdf]

Supplementary Text S1. Standard Solutions and Calibration

S1.1. Standards and Reagents

The mixed phthalate standard solution (1000 μg/mL) contained DBP (99.4% purity) and DEHP (98.0% purity). The mixed isotope-labeled internal standard stock solution (100 μg/mL) contained D4-DBP and D4-DEHP (99.0% purity). Both were obtained from BePure (China) and stored at 0-4 °C in the dark.

S1.2. Preparation of Working Solutions

Intermediate standard solution (10 μg/mL): 1 mL of the mixed standard solution (1000 μg/mL) was diluted to 100 mL with n-hexane.

Internal standard working solution (5 μg/mL each): 5 mL of the isotope-labeled IS stock solution (100 μg/mL) was diluted to 100 mL with n-hexane.

S1.3. Calibration Standards

Calibration working solutions were prepared freshly by serially diluting the intermediate solution (10 μg/mL) to concentrations of 0, 0.01, 0.02, 0.05, 0.10, 0.20, 0.50, and 1.00 μg/mL. Each calibration standard contained the internal standard at a fixed concentration of 0.100 μg/mL. Note: In sample analysis, adding 40 μL of IS working solution (5 μg/mL) to a sample that is finally reconstituted in 2.0 mL results in a final IS concentration of 0.100 μg/mL, matching the calibration standards.

Supplementary Text S2. GC-MS Parameters

S2.1. GC Conditions

Carrier gas: helium (1.0 mL/min). Injection: 1 μL, splitless, inlet at 250 °C. Oven program: 60 °C (1 min), ramp to 220 °C at 20 °C/min (1 min), ramp to 280 °C at 5 °C/min (5 min).

S2.2. MS Conditions

Source: 230 °C; quadrupole: 150 °C; transfer line: 280 °C. Mode: EI (70 eV), SIM. Solvent delay: 6 min.

Supplementary Text S3. Method Validation Results

S3.1. Precision and Accuracy

Method accuracy was assessed using spike-recovery experiments. Samples were spiked at three levels: DBP: 0.15, 0.30, and 1.50 mg/kg. DEHP: 0.30, 0.60, and 1.50 mg/kg. Across the study, spike recoveries ranged from 70% to 117%. Method precision was evaluated through duplicate analyses ($n \geq 2$).

S3.2. QC Material Monitoring

The certified QC material (103CO02055) was analyzed in each batch. Measured values were consistently within the certified ranges: 2.59-2.66 mg/kg for DBP (Certified: 2.462-3.002 mg/kg) and 1.34-1.53 mg/kg for DEHP (Certified: 1.326-1.982 mg/kg).

Table S1. Retention times and monitoring ions for target compounds and internal standards.

Compound	Retention Time (RT, min)	Qualitative Ions (m/z)	Quantitative Ion (m/z)
D4-DBP	11.122	153, 227, 209, 108	153
DBP	11.137	149, 223, 205, 104	149
D4-DEHP	17.528	153, 171, 283, 117	153
DEHP	17.550	149, 167, 279, 113	149

Table S2. Maximum permitted tolerances for relative ion intensities in GC-MS qualitative confirmation (Reference: Commission Decision 2002/657/EC)[1].

Relative Abundance, % of Base Peak	Maximum Permitted Tolerance for GC-MS
>50%	±10%
20%-50%	±15%
10%-20%	±20%
≤10%	±50%

Table S3. The fitted parameters of the traditional parametric models for positive concentrations of DEHP and DBP.

**Contaminant**	**Distribution**	**Parameter 1**	**Parameter 2**
**DEHP**	Lognormal	-1.31	0.84
	Weibull	0.77	0.44
	Gamma	0.79	1.34
	Exponential	/	1.72
**DBP**	Lognormal	-1.83	0.76
	Weibull	1.23	0.24
	Gamma	1.69	7.64
	Exponential	/	4.51

For the Lognormal distribution, Parameters 1 and 2 are the mean and standard deviation of the log-transformed data, respectively. For Weibull and Gamma distributions, they represent the shape and scale parameters. For the Exponential distribution, Parameter 2 is the rate parameter (λ), and Parameter 1 is not applicable (/).

Table S4. The fitted parameters of the extreme value mixture model for positive concentrations of DEHP and DBP.

Contaminant	Distribution	Parameter 1	Parameter 2	Threshold	Parameter 3	Parameter 4
DEHP	Lognormal-GPD	-1.43	0.55	0.4	0.35	0.80

	Weibull-GPD	2.04	0.30	0.4	0.35	0.80
	Gamma-GPD	3.77	0.07	0.4	0.35	0.80
DBP	Lognormal-GPD	-1.83	0.78	0.5	0.53	-0.99
	Weibull-GPD	1.23	0.24	0.5	0.53	-0.99
	Gamma-GPD	1.69	0.13	0.5	0.53	-0.99

The Extreme value mixture model consist of a bulk distribution for data below a threshold (u) and a generalized Pareto distribution (GPD) for data above it. For the bulk component (Lognormal, Weibull, or Gamma), Parameters 1 and 2 are the respective primary parameters (i.e., log-mean/log-sd, shape/scale). For the tail component, Parameter 3 is the GPD scale parameter (σ) and Parameter 4 is the GPD shape parameter (ξ).

Table S5. The fitted parameters of the mixture model for positive concentrations of DEHP.

Distribution	Parameter 1	Parameter 2	Mixing ratio	Parameter 3	Parameter 4
2-Lognormal	-1.6564	0.3774	0.699：0.301	-0.4980	1.04763
2-Weibull	1.4295	0.3504	0.98：0.02	160.00	13.66
2-Gamma	4.3980E+13	3.2197E+12	0.02：0.98	2.3203	7.3802

This table presents the parameters for 2-component mixture models. The 'Mixing ratio' ($\pi1{:}\pi2$) indicates the estimated proportion of each component. Parameters 1 & 2 define the first component, while Parameters 3 & 4 define the second component. For the 2-Lognormal model, the parameters are log-mean and log-standard deviation. For the 2-Weibull model, they are shape and scale parameters. For the 2-Gamma model, they are the shape and rate parameters for each component.

Table S6. Hazard quotients (HQs) and 95% confidence intervals (CIs) for exposure to DEHP and DBP from hot pot bases in the Chinese population

**Contaminants**	**Group**	**HQs(95%CI)**				
		**Mean**	**P50**	**P75**	**P95**	**P99.9**
DEHP	M,7-13yrs	0.0080 (0.0072, 0.0087)	0.0022 (0.0021, 0.0023)	0.0056 (0.0054, 0.0058)	0.0195 (0.0186, 0.0203)	0.6548 (0.5566, 0.7312)
	F,7-13yrs	0.0077 (0.0071, 0.0084)	0.0020 (0.0019, 0.0021)	0.0052 (0.0050, 0.0054)	0.0196 (0.0187, 0.0205)	0.6805 (0.5706, 0.7670)
	M,14-17yrs	0.0061 (0.0054, 0.0067)	0.0017 (0.0017, 0.0018)	0.0044 (0.0042, 0.0045)	0.0146 (0.0139, 0.0153)	0.4854 (0.4061, 0.5470)
	F,14-17yrs	0.0060 (0.0054, 0.0066)	0.0016 (0.0015, 0.0016)	0.0041 (0.0040, 0.0043)	0.0150 (0.0144, 0.0158)	0.5084 (0.4314, 0.5746)
	M,18-49yrs	0.0064 (0.0058, 0.0069)	0.0018 (0.0017, 0.0018)	0.0045 (0.0044, 0.0047)	0.0152 (0.0146, 0.0158)	0.5103 (0.4461, 0.5645)
	F,18-49yrs	0.0063 (0.0056, 0.0069)	0.0017 (0.0017, 0.0018)	0.0045 (0.0043, 0.0046)	0.0152 (0.0146, 0.0160)	0.5151 (0.4505, 0.5817)
	M,50-75yrs	0.0065 (0.0059,	0.0019 (0.0018,	0.0047 (0.0045,	0.0151 (0.0145,	0.4991 (0.4233,

Contaminants	Group	HQs(95%CI)				
		Mean	P50	P75	P95	P99.9
		0.0070)	0.0019)	0.0049)	0.0157)	0.5526)
	F,50-75yrs	0.0063 (0.0058, 0.0069)	0.0018 (0.0017, 0.0018)	0.0045 (0.0044, 0.0047)	0.0152 (0.0145, 0.0160)	0.5116 (0.4625, 0.5541)
DBP	M,7-13yrs	0.0100 (0.0096, 0.0104)	0.0035 (0.0034, 0.0036)	0.0089 (0.0086, 0.0092)	0.0416 (0.0392, 0.0439)	0.2201 (0.1933, 0.2524)
	F,7-13yrs	0.0096 (0.0092, 0.0101)	0.0032 (0.0031, 0.0033)	0.0085 (0.0082, 0.0088)	0.0404 (0.0380, 0.0426)	0.2232 (0.1947, 0.2537)
	M,14-17yrs	0.0077 (0.0074, 0.0080)	0.0027 (0.0026, 0.0028)	0.0069 (0.0066, 0.0071)	0.0318 (0.0301, 0.0336)	0.1642 (0.1455, 0.1876)
	F,14-17yrs	0.0075 (0.0072, 0.0078)	0.0025 (0.0024, 0.0026)	0.0066 (0.0064, 0.0069)	0.0314 (0.0297, 0.0331)	0.1721 (0.1505, 0.1980)
	M,18-49yrs	0.0080 (0.0076, 0.0082)	0.0028 (0.0027, 0.0029)	0.0071 (0.0069, 0.0073)	0.0328 (0.0312, 0.0347)	0.1728 (0.1538, 0.1993)
	F,18-49yrs	0.0079 (0.0076, 0.0081)	0.0028 (0.0026, 0.0028)	0.0070 (0.0068, 0.0072)	0.0327 (0.0310, 0.0341)	0.1722 (0.1530, 0.1944)
	M,50-75yrs	0.0081 (0.0077, 0.0084)	0.0029 (0.0028, 0.0030)	0.0073 (0.0071, 0.0075)	0.0332 (0.0313, 0.0353)	0.1665 (0.1488, 0.1858)
	F,50-75yrs	0.0079 (0.0076, 0.0082)	0.0028 (0.0027, 0.0029)	0.0071 (0.0068, 0.0073)	0.0327 (0.0309, 0.0347)	0.1691 (0.1508, 0.1931)

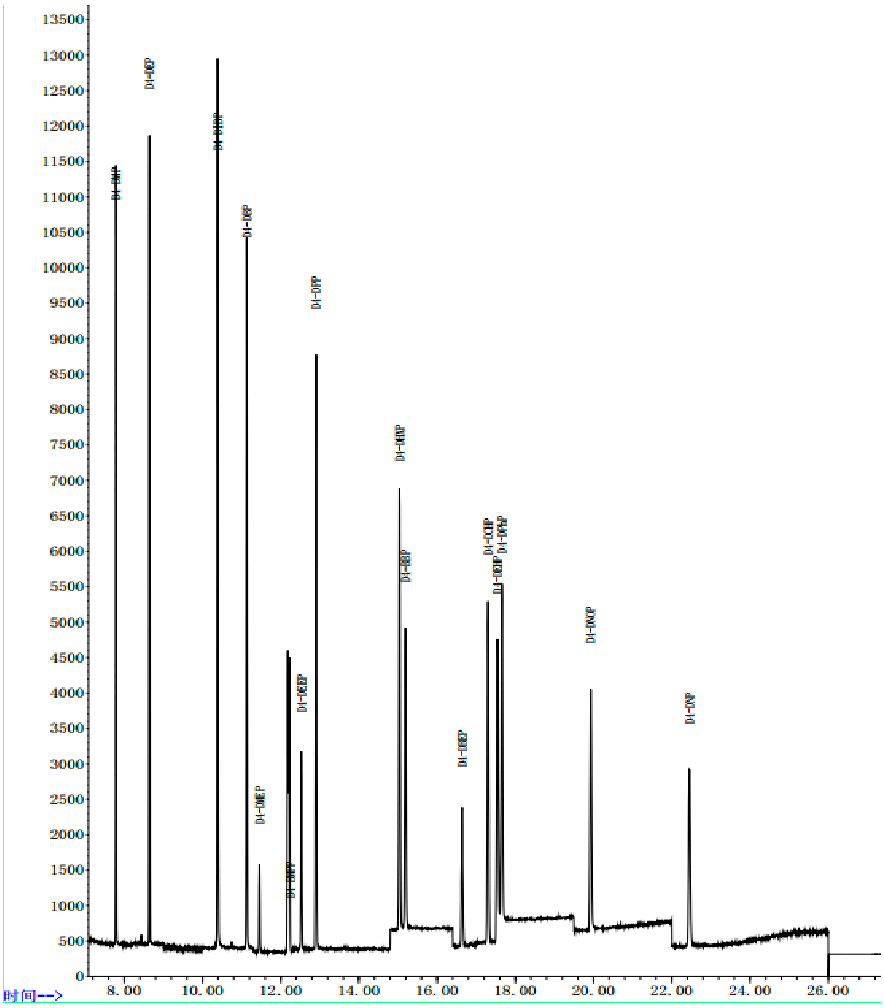

Figure S1. Total ion chromatogram (TIC) of a procedural blank processed using the QuEChERS–GC–MS method.

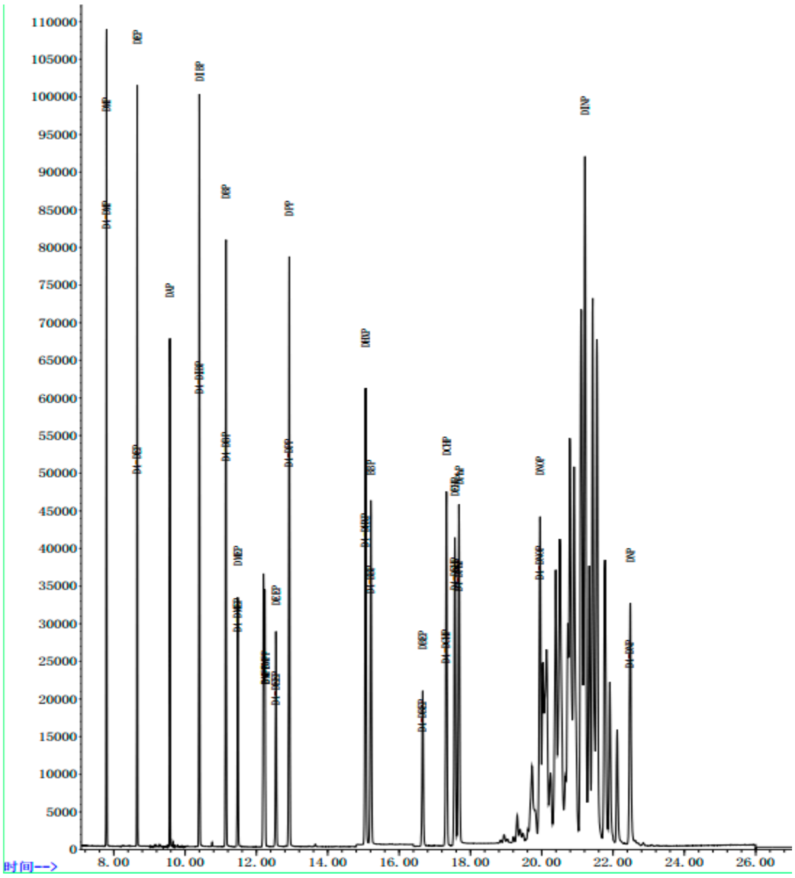

Figure S2. Total ion chromatogram (TIC) of the mixed PAE standard solution under the optimized GC–MS conditions.

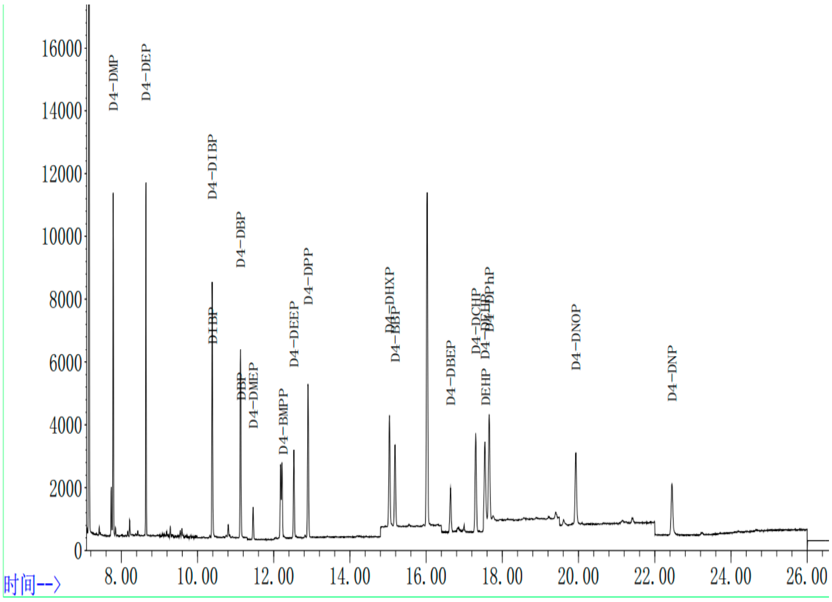

Figure S3. Representative total ion chromatogram (TIC) of a hot pot soup base sample under the optimized GC–MS conditions.

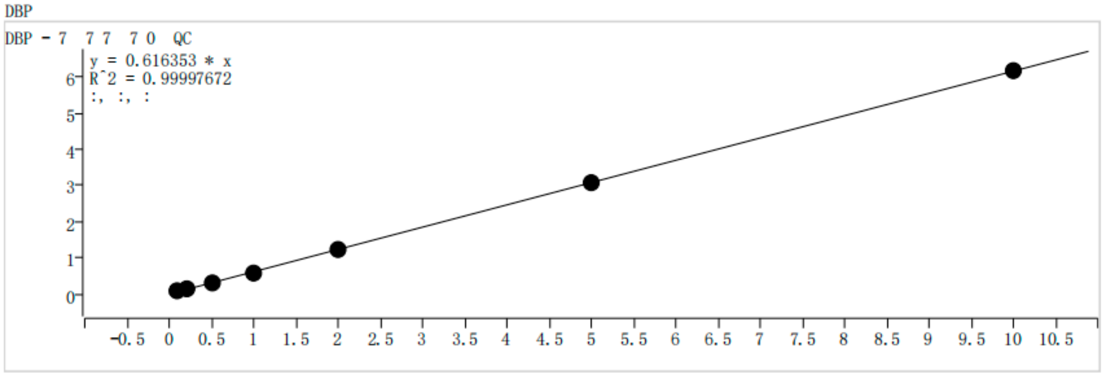

Figure S4. Calibration curve for DBP obtained by isotope-dilution GC–MS (multi-point external calibration with internal-standard correction).

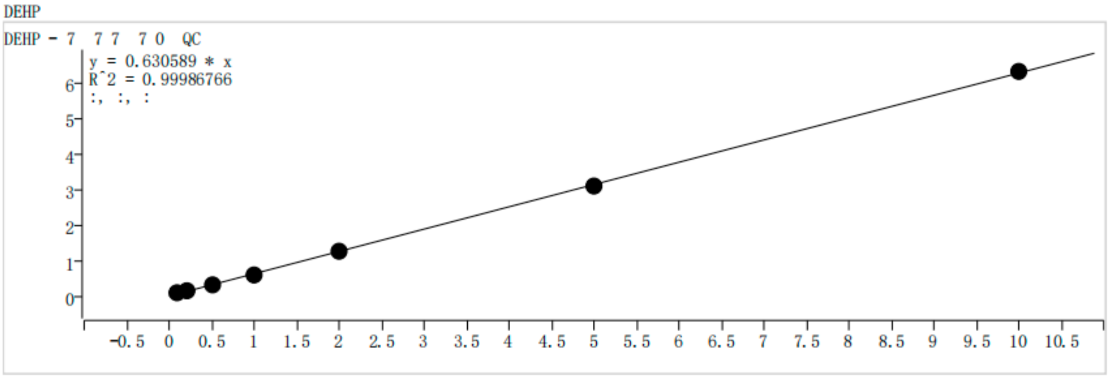

Figure S5. Calibration curve for DEHP obtained by isotope-dilution GC–MS (multi-point external calibration with internal-standard correction).

References

1. European Commission. Commission Decision 2002/657/EC implementing Council Directive 96/23/EC concerning the performance of analytical methods and the interpretation of results. Off. J. Eur. Commun. 2002, 221, 8–36.
